# Low Serum Fetuin-A as a Biomarker to Predict Pneumococcal Necrotizing Pneumonia and Hemolytic Uremic Syndrome in Children: Erratum

**DOI:** 10.1097/MD.0000000000009684

**Published:** 2018-01-19

In the article, “Low Serum Fetuin-A as a Biomarker to Predict Pneumococcal Necrotizing Pneumonia and Hemolytic Uremic Syndrome in Children”,^[1]^ which appeared in Volume 95, Issue 13 of *Medicine*, the supplemental digital content link, is missing.

## Supplementary Material

Supplemental Digital Content
