# Supplementary material for: Low Serum Fetuin-A as a Biomarker to Predict Pneumococcal Necrotizing Pneumonia and Hemolytic Uremic Syndrome in Children: Erratum
Source: Medicine (Baltimore). 2018 Jan 19;97(3):e9684. doi: 10.1097/MD.0000000000009684 (PMC5779789; doi:10.1097/MD.0000000000009684)
Supplement: Supplemental Digital Content [file medi-97-e9684-s001.pdf]

Supplemental Digital Content

Materials and methods

Protein Expression and Purification

S. pneumoniae strain CGSP14 is a serotype 14 clinical isolate derived from a child with HUS. PCR fragments containing the protein coding sequences of NanA, NanB, and NanC were amplified using genomic DNA from CGSP14. Neuraminidase gene *nanA* (2941 bp) was amplified by primers 5'-AGATCTGGGTACCATGTCTTATTTCAGAAATCG-3' and 5'- TGGTGCTCGAGTTGTTCTCTCTTTTTCCCTA-3', *nanB* (2091 bp) by 5'- AGATCTGGGTACCATGAATAAAAGAGGTCTTTA-3' and 5'- TGGTGCTCGAGTTTTGTTAAATCATTAATTTC-3', and *nanC* (2220 bp) by 5'- AGATCTGGGTACCATGAAAAAAAATATTAAACA-3' and 5'- TGGTGCTCGAGATTCTTTTTCAGATCTTCAA-3'. Amplified fragments were cloned, with the resulting plasmids transformed into *E. coli* DH5α.

Pneumococcal Neuraminidase Substrate Specificity Assay

Lectins are widely used to recognize glycoconjugate residues on cells. Fluorescein-labeled peanut agglutinin (PNA) was used to detect TA on cells; fluorescein-labeled *Sambucus Nigra* lectin (SNA) and biotinylated *Maackia Amurensis* lectin II (MAL II) were used to recognize α2-6 and α2-3 sialyl linkages, respectively.

To detect the glycoconjugates on RBC, freshly collected blood samples from healthy volunteers were used to prepare the RBC fraction. RBC

(10^6 - 10^7 cells/mL) were treated with NanA, NanB or NanC and incubated for 1-2 hours at 37°C. Labeling was done at 4°C with lectins PNA, SNA and MAL II and flow cytometric (FACScan, Becton Dickinson, USA) analysis was performed using 10,000-20,000 cells. Biotinylated MAL II labeling was detected with fluorescein conjugated streptavidin. Different concentrations of bovine fetuin (Sigma, St. Louis, MO, USA) were added to the above assay to analyze the protective role of fetuin.

Cleavage specificity of pneumococcal neuraminidases NanA, NanB and NanC was also determined as described earlier using the Amplex Red neuraminidase (sialidase) assay kit (Invitrogen, Carlsbad, CA, USA). 3'-Sialyllactose and 6'-Sialyllactose were used as α2-3 and α2-6 linked sialic acid substrates, respectively (Sigma-Aldrich, St. Louis, MO, USA).

Human Serum Sialoglycoproteins Profiling

Neuraminidase (NanA), beta-galactosidase (BgaA), and a beta-N-acetylglucosaminidase (StrH) act sequentially to remove sialic acid, galactose and N-acetylglucosamine and expose mannose on human glycoproteins; NanA cleaves sialic acid residues on cells and circulating sialoglycoproteins leading to the exposure of the TA (galactosyl (β-1,3) N-acetylgalactosamine). Lectins are widely used to recognize glycoconjugate residues on various cells. Peanut agglutinin (PNA) binds preferentially to Thomsen-Friedenrich antigen (T-antigen; TA). Briefly, serum from normal and healthy adults was diluted in PBS (pH = 8.0), and treated with NanA, NanB and NanC. Later all the samples were biotinylated with EZ-link sulfo-NHS-LC-LC-Biotin (Pierce, Rockford, IL, USA). To capture asialoglycoproteins, samples were incubated with

PNA-conjugated agarose and eluted with lactose in Tris-buffered saline. The samples were then separated on SDS-polyacrylamide gels (1D) and transferred to Immobilon P membranes (Millipore, Billerica, MA, USA). The membranes were washed and then probed with streptavidin-horse radish peroxidase (R&D Systems Inc. Minneapolis, MN, USA.). Proteins were visualized by chemiluminescence (Perkin-Elmer Life Sciences, Boston, MA, USA). And protein samples were submitted to liquid chromatography-tandem mass spectrometry (LC-MS/MS) analysis.

Trypsin Digestion and Sample Clean-up

The in-solution digestion of each protein mixture was performed in 50 mM NH_4HCO_3. Each sample was reduced by 5 mM DTT at 56℃ for 30 min and alkylated by 15mM iodoacetamide at room temperature for 30 min. Unreacted iodoacetamide was quenched by another 5 mM DTT at room temperature for 30 min. The solution was diluted five-fold with 10 mM NH_4HCO_3, and digestion was performed in the presence of 100 ng trypsin (Promega, Madison WI, USA) at 37℃ overnight. The resulting peptide mixture was clean-up by a SOURCE 30S (*GE Healthcare)* strong cation exchange microcolumn. Peptides were eluted by 0.1M NH_4HCO_3 containing 0.5 M NaCl and 30% acetonitrile. After dried with a SpeedVac concentrator, the sample was reconstituted with 5% formic acid/H_2O and subjected to LC-MS/MS analysis.

Nano-LC-tandem Mass Spectrometry

A Surveyor nano-LC system connected with a LTQ-Orbitrap mass spectrometer (Thermo Scientific, Bremen, Germany) was used in this study.

Mobile phase A and B used for the LC system were 0.1% formic acid/H2O and 0.1% formic acid/acetonitrile, respectively. The peptide solution (6.4 μL) was loaded into the reverse phase trap column (ZORBAX 300SB-C18, 0.3 x 5 mm, Agilent) with mobile phase A at a flow rate of 6 μL /min for 7 min. The trap column was then switched to a BioBasic C18 analytical column (75 μm i.d.× 15 cm, New Objective). Peptides were eluted at a flow rate of 300 nL/min with the following gradient: 0–40 min, 5–30% B; 40-45 min, 30–45% B; 45-47 min, 45-95% B; 47–51 min, 95% B; 51–53 min, 95-5% B; and 53–65 min, 5% B. The LTQ-Orbitrap equipped with a nanoelectrospray ion source (Thermo Scientific) was operated in positive ion mode. The spray voltage was set to 1.8 kV. Full-scan MS spectra (m/z 400-m/z 2000) were acquired in the Orbitrap mass analyzer at a resolution of 30,000 at m/z 400. The m/z 445.1200, 462.1466, and 536.1654 cyclosiloxane peaks were used for lock mass calibration to improve mass accuracy. For the information dependent acquisition, eight most intense ions with a minimal signal intensity of 10,000 were selected for collision induced fragmentation (CID) in the linear ion trap with the following parameter settings: isolation width of 2.0, normalized collision energy of 39%, activation Q of 0.25, and activation time of 30 ms. Each precursor ion was allowed to be sequenced twice and then excluded dynamically for 75 seconds.

Database Searching

RAW files were analyzed with Proteome Discoverer 1.3 software (Thermo Scientific). The CID spectra were searched against the UniProt human database (June 2010, 20294 sequences) through Mascot search engine. The search parameters were as follows: enzyme specificity was trypsin; a

maximum of two miscleavages was allowed; precursor mass tolerance was 10 ppm; fragment mass tolerance was 0.6 Da; acetylation at protein N-terminus, Gln to pyroglutamic acid conversion at peptide N-terminus, and methionine oxidation were set as variable modifications; and cysteine carbamidomethylation was set as a fixed modification. False discovery rate (FDR) of peptide/protein identifications were determined by employing a decoy database searching. Only high confident peptide/protein identifications (FDR<1%) were considered for further evaluation. The search result was sent to the ProteinCenter server (Thermo Scientific) for functional annotation.

Fetuin RBC Protection Assay

To detect the exposure of TA on red blood cells (RBC), freshly collected blood samples from healthy volunteers were used to prepare the RBC fraction. Red blood cells (RBC) (3 x 10^7 cells/mL) were treated with NanA, NanB or NanC and incubated for 1-2 hours at 37°C. Labeling was done at 4°C with fluorescein-PNA (Vector Laboratories, Burlingame, CA, USA) and flow cytometric analysis (FACScan, Becton Dickinson, USA) was performed using 10,000-20,000 cells. Different concentrations of bovine fetuin (Sigma, St. Louis, MO, USA) were added to the above assay to analyze the protective role of fetuin.

Enzyme-linked Immunosorbent Assay (ELISA)

Serum fetuin-A levels from patients and controls were determined by a sandwich ELISA (Human fetuin-A ELISA kit, R&D Systems,

Minneapolis, MN, USA). Serum fetuin-A levels were determined by a prepared calibration curve. The mean minimum detectable dose of this assay system is 0.62 ng/mL (range, 0.16-1.74 ng/mL). When results fell outside of the range, samples were diluted, retested, and multiplied by the dilution factor.

Supplemental Figure S1. Activity and sialic acid linkages specificity of NanA, NanB and NanC by flow cytometry. (A) Thomsen-Friedenrich antigen (TA) exposure on cells. NanA, NanB and NanC (0.1 μg) can expose TA on RBC. PNA lectin was used to detect the TA. (B**)** Only NanA cleaves α2-6 linkages on RBC, SNA lectin binding was used to detect the α2-6 linkages by flow cytometry. (C) NanA, NanB and NanC showed activity against α2-3 linkages on RBC. MAL II lectin binding was used to detect the α2-3 linkages on RBC. Numbers at the top of the panel indicate fluorescence counts: black (untreated cells), red (NanA-treated), blue (NanB-treated), green (NanC-treated).

A

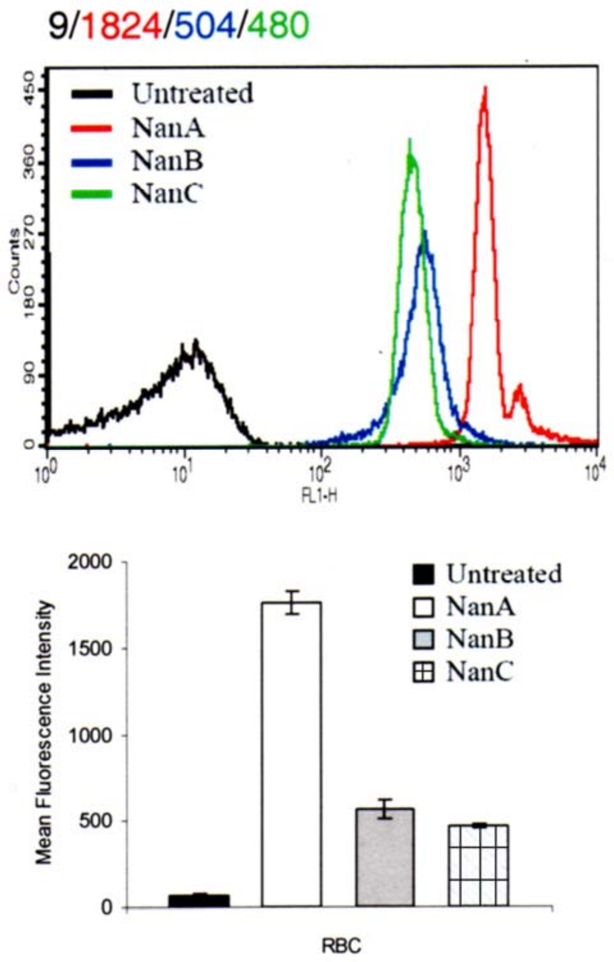
9/1824/504/480
Untreated
NanA
NanB
NanC
Counts
0
90
180
270
360
450
FL1-H
Mean Fluorescence Intensity
2000
1500
1000
500
0
RBC

B

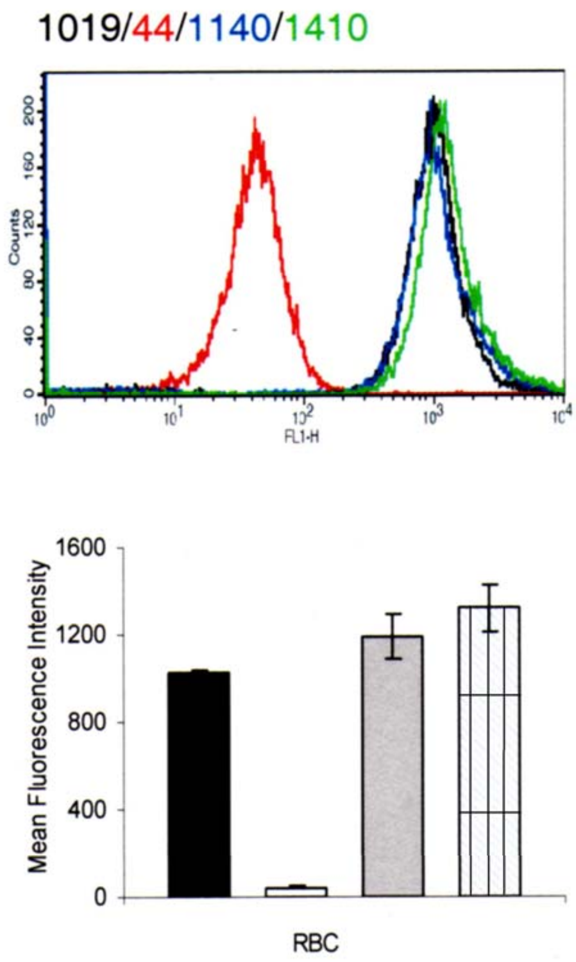
1019/44/1140/1410
Counts
FL1-H
Mean Fluorescence Intensity
1600
1200
800
400
0
RBC

C

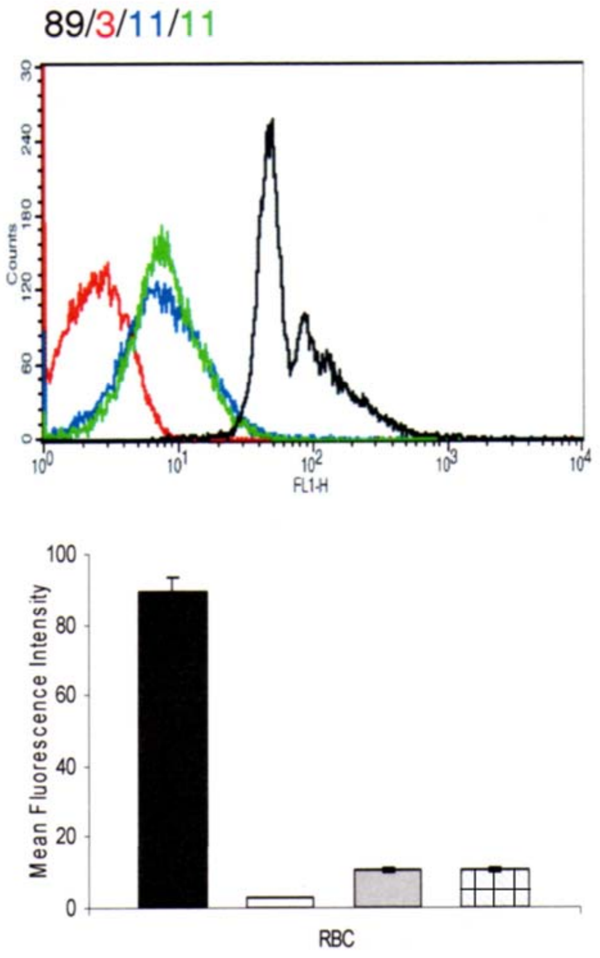

Supplemental Figure S2. Substrate specificity of NanA, NanB and NanC using the Amplex Red neuraminidase (sialidase) assay kit. (A) Activity against α2-6 linkage and (B) Activity against α2-3 linkage, and. Fetuin was replaced with α2-3, α2-6 sialyllactoses to analyze α2-3 and α2-6 sialic acid link specificity. (C) NanA, NanB and NanC activity. Bovine fetuin was used as a substrate. C-Neu is the *Clostridium perfringens* neuraminidase provided in the kit and used as control (0.1-0.5 U/mL).

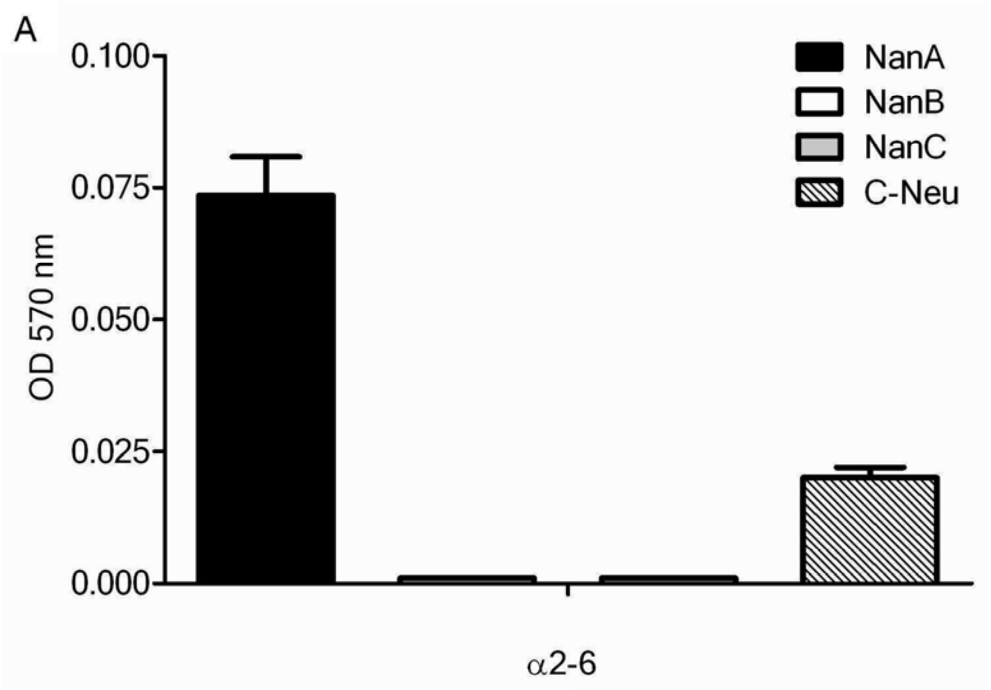

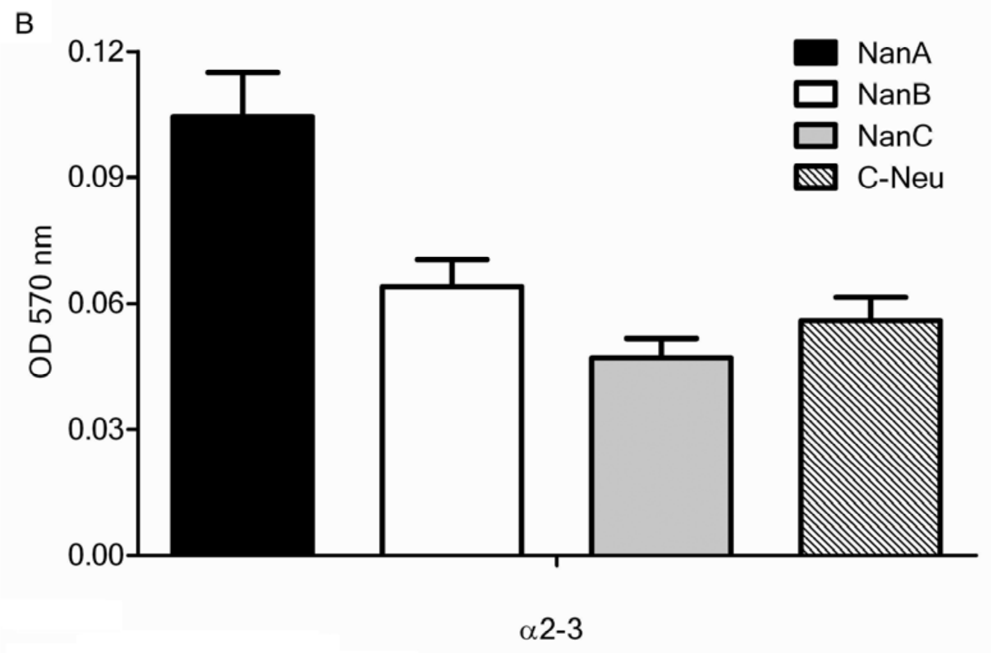
B
0.12
0.09
0.06
0.03
0.00
OD 570 nm
NanA
NanB
NanC
C-Neu
α2-3

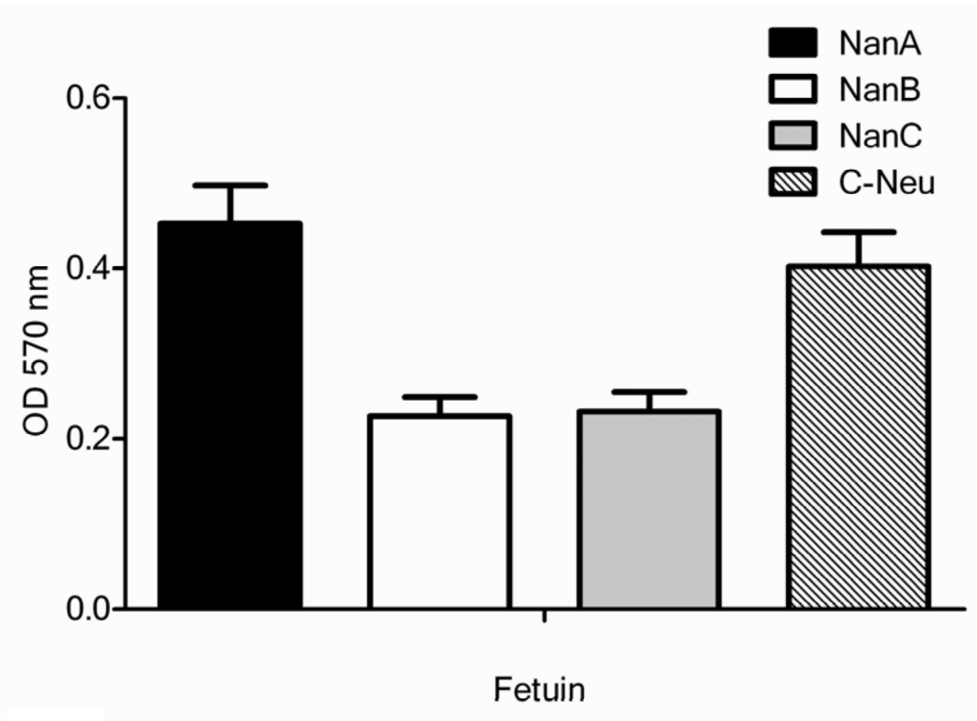
NanA
NanB
NanC
C-Neu
0.6
0.4
0.2
0.0
OD 570 nm
Fetuin

Supplemental Figure S3. NanA, NanB and NanC differentially cleave serum sialoglycoproteins. (A) Western blot of biotinylated asialoglycoproteins after NanA, NanB and NanC treatment and capture by peanut lectin column. (B) Western blot with anti-fetuin A antibody to detect asialo-fetuin-A captured by peanut lectin from serum after NanA, NanB and NanC treatment (left) and to detect fetuin-A in total serum (right). UT denotes untreated serum control.

A

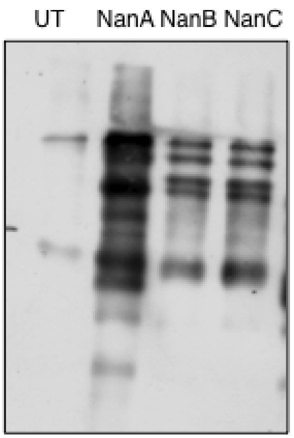

B

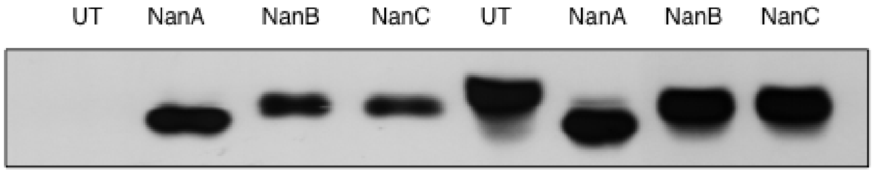

Supplemental Figure S4. Serum fetuin-A concentration during acute stage of infection and recovery. Consecutive blood samples were collected from the patient with pneumococcal meningitis and serum fetuin-A concentration was determined by ELISA.

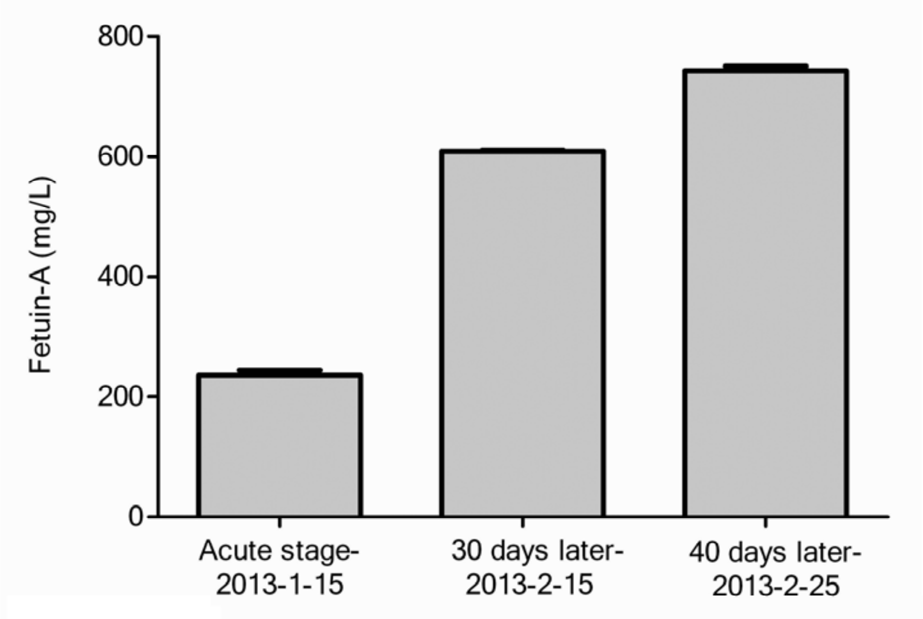

Supplemental Table S1. T antigen containing glycoproteins in untreated and neuraminidase-treated serum identified by LC/MS after binding to peanut lectin-conjugated agarose column.

Neuraminidase treatment	Protein Name	Molecular Mass [kDa]	Accession Number	Matching Peptides	Sequence Coverage
None	Serum albumin	69.3	P02768	17	31.36
	Ig alpha-1 chain C region	37.6	P01876	9	31.44
	Keratin, type II cytoskeletal 1	66.0	P04264	9	24.53
	Alpha-amylase 1	57.7	P04745	8	19.37
	Keratin, type I cytoskeletal 10	58.8	P13645	7	13.18
	Neutrophil defensin 1	10.2	P59665	3	20.21
	Keratin, type I cytoskeletal 9	62.0	P35527	3	8.35
	Keratin, type II cytoskeletal 2	65.4	P35908	3	7.04

	epidermal				
	Ig lambda chain C regions	11.2	P01842	2	21.90
	Hemoglobin subunit alpha	15.2	P69905	2	21.83
	Cystatin-S OS=Homo sapiens	16.2	P01036	2	20.57
	Lysozyme C OS=Homo sapiens	16.5	P61626	2	14.19
	Actin, cytoplasmic 1 OS=Homo sapiens	41.7	P60709	2	7.73
	Lactotransferrin OS=Homo sapiens	78.1	P02788	2	3.10
	Fibrinogen alpha chain	94.9	P02671	2	3.35
	Serum albumin	69.3	P02768	47	75.86
	Inter-alpha-trypsin inhibitor heavy chain H4	103.3	Q14624	27	38.49
	Ig alpha-1 chain C region	37.6	P01876	25	66.01
	Apolipoprotein B-100	515.2	P04114	23	7.06
	Inter-alpha-trypsin inhibitor heavy	106.4	P19823	20	29.49

	chain H2				
	Inter-alpha-trypsin inhibitor heavy chain H1	101.3	P19827	20	33.37
	Fibrinogen gamma chain	51.5	P02679	20	45.25
	Fibrinogen alpha chain	94.9	P02671	20	26.10
	Fibrinogen beta chain	55.9	P02675	19	44.40
NanA	Fibronectin	262.4	P02751	18	11.44
	Keratin, type II cytoskeletal 1	66.0	P04264	13	40.06
	Hemopexin	51.6	P02790	13	37.88
	Vitamin D-binding protein	52.9	P02774	12	31.86
	Kininogen-1	71.9	P01042	12	22.67
	**Alpha-2-HS-glycoprotein (Fetuin –A)**	**39.3**	**P02765**	**11**	**26.43**
	Keratin, type II cytoskeletal 2 epidermal	65.4	P35908	11	26.45

	Keratin, type I cytoskeletal 10	58.8	P13645	10	19.01
	Ig lambda chain C regions	11.2	P01842	8	97.14
	Ig kappa chain C region	11.6	P01834	8	93.40
	Plasma protease C1 inhibitor	55.1	P05155	8	18.00
	Histidine-rich glycoprotein	59.5	P04196	8	14.86
	Vitronectin	54.3	P04004	7	24.69
	Plasminogen	90.5	P00747	7	13.58
	Alpha-2-macroglobulin	163.2	P01023	7	6.11
	Keratin, type I cytoskeletal 9	62.0	P35527	6	19.10
	Protein AMBP	39.0	P02760	6	16.48
	Apolipoprotein E	36.1	P02649	5	18.93
	Immunoglobulin J chain	18.1	P01591	5	26.42
	Complement C1r subcomponent	80.1	P00736	5	11.63
	Coagulation factor XII	67.8	P00748	5	11.71
	Complement factor B	85.5	P00751	5	7.98

Clusterin	52.5	P10909	4	10.69
Complement C1s subcomponent	76.6	P09871	4	6.69
Ig mu chain C region	49.3	P01871	4	16.59
N-acetylmuramoyl-L-alanine amidase	62.2	Q96PD5	4	15.80
C4b-binding protein alpha chain	67.0	P04003	4	10.39
Ig gamma-2 chain C region	35.9	P01859	3	24.85
Ig gamma-3 chain C region	41.3	P01860	3	24.93
Hornerin	282.2	Q86YZ3	3	5.26
Apolipoprotein A-II	11.2	P02652	3	39.00
Extracellular matrix protein 1	60.6	Q16610	2	5.56
Complement C3	187.0	P01024	2	2.53
Serotransferrin	77.0	P02787	2	2.87
Ig lambda chain V-III region LOI	11.9	P80748	2	15.32
Alpha-1-antitrypsin	46.7	P01009	2	4.31
Complement component C9	63.1	P02748	2	3.94

	Adiponectin	26.4	Q15848	2	15.16
	von Willebrand factor	309.1	P04275	2	1.03
NanC	Serum albumin	69.3	P02768	28	55.01
	Keratin, type II cytoskeletal 1	66.0	P04264	16	37.11
	Ig alpha-1 chain C region	37.6	P01876	13	41.36
	Kininogen-1	71.9	P01042	12	24.69
	Inter-alpha-trypsin inhibitor heavy chain H4	103.3	Q14624	11	15.05
	Keratin, type I cytoskeletal 10	58.8	P13645	9	20.21
	Ig lambda chain C regions	11.2	P01842	6	76.19
	Inter-alpha-trypsin inhibitor heavy chain H1	101.3	P19827	6	9.44
	Fibrinogen alpha chain	94.9	P02671	6	11.66
	Keratin, type II cytoskeletal 2 epidermal	65.4	P35908	5	15.34

Inter-alpha-trypsin inhibitor heavy chain H2	106.4	P19823	5	8.25
Ig kappa chain C region	11.6	P01834	5	63.21
Fibrinogen beta chain	55.9	P02675	5	15.68
Hepatocyte growth factor activator	70.6	Q04756	5	12.06
**Alpha-2-HS-glycoprotein (Fetuin –A)**	**39.3**	**P02765**	**5**	**19.62**
Apolipoprotein B-100	515.2	P04114	4	1.29
Keratin, type I cytoskeletal 9	62.0	P35527	3	8.35
Fibrinogen gamma chain	51.5	P02679	3	5.52
Complement C1r subcomponent	80.1	P00736	3	5.39
Plasma protease C1 inhibitor	55.1	P05155	3	12.00
Hemopexin	51.6	P02790	3	10.17
Apolipoprotein E	36.1	P02649	3	11.04
Protein AMBP	39.0	P02760	2	6.53

	Immunoglobulin J chain	18.1	P01591	2	13.84
	Keratin, type II cytoskeletal 5	62.3	P13647	2	5.59
	Complement C1s subcomponent	76.6	P09871	2	5.09
	Coagulation factor XII	67.8	P00748	2	6.18
	Fibronectin	262.4	P02751	2	1.59

Supplemental Table S2. Identification of asialofetuin binding to peanut lectin column by LC/MS.

Neuramindase	Protein Name	Molecular Mass (kDa)	Accession Number	Matching Peptides	Sequence Coverage (%)	Glycosylation	
						N	O
NanA	Fetuin –A	39.3	P02765	11	26	+	+
NanC	Fetuin –A	39.3	P02765	5	20	+	+

Abbreviation: N = N-glycosylation; O = O-glycosylation
